# Evaluation of Surgical Therapy in Advanced Thymic Tumors

**DOI:** 10.3390/cancers13184516

**Published:** 2021-09-08

**Authors:** Till Markowiak, Mohammed Khalid Afeen Ansari, Reiner Neu, Berthold Schalke, Alexander Marx, Hans-Stefan Hofmann, Michael Ried

**Affiliations:** 1 Department of Thoracic Surgery, University Medical Center Regensburg, 93053 Regensburg, Germany; till.markowiak@ukr.de (T.M.); mohammed-khalid-afeen.ansari@ukr.de (M.K.A.A.); reiner.neu@ukr.de (R.N.); hans-stefan.hofmann@ukr.de (H.-S.H.); 2 Department of Neurology, University Medical Center Regensburg, 93053 Regensburg, Germany; Berthold.Schalke@medbo.de; 3 Institute of Pathology, University Medical Center Mannheim, University of Heidelberg, 68167 Mannheim, Germany; Alexander.Marx@umm.de; 4 Department of Thoracic Surgery, Hospital Barmherzige Brüder Regensburg, 93049 Regensburg, Germany

**Keywords:** thymic tumor, thymoma, thymic carcinoma, hyperthermic intrathoracic chemotherapy

## Abstract

**Simple Summary:**

A complete resection of thymic tumors is known to be the most important prognostic factor, but it is often difficult to perform, especially in advanced stages. In this study, patients with advanced thymic tumors who underwent radical resection were examined retrospectively. The primary endpoint was defined as the postoperative resection status. Secondary endpoints included postoperative morbidity, mortality, and survival. In tumor stages III a microscopic complete resection was achieved in 53.3% of patients. In stages IV a macroscopic complete resection was documented in 76.7% of patients. Surgical revision was necessary in 17.8% of patients. In-hospital mortality was 2.7%. The 5-year survival rate of all patients was 61.3%. In particular, median survival after macroscopic incomplete resection was significantly short. Advanced thymic tumors can be resected with an acceptable risk of complications and low mortality. In stage III as well as in stage IV the promising survival rates are dependent on the resection-status.

**Abstract:**

A complete resection of thymic tumors is known to be the most important prognostic factor, but it is often difficult to perform, especially in advanced stages. In this study, 73 patients with advanced thymic tumors of UICC stages III and IV who underwent radical resection were examined retrospectively. The primary endpoint was defined as the postoperative resection status. Secondary endpoints included postoperative morbidity, mortality, recurrence/progression-free, and overall survival. In total, 31.5% of patients were assigned to stage IIIa, 9.6% to stage IIIb, 47.9% to stage IVa, and 11% to stage IVb. In stages III a R0 resection was achieved in 53.3% of patients. In stages IV a R0/R1 resection was documented in 76.7% of patients. Surgical revision was necessary in 17.8% of patients. In-hospital mortality was 2.7%. Median recurrence/progression-free interval was 43 months (*p* = 0.19) with an overall survival of 79 months. The 5-year survival rate was 61.3%, respectively. Median survival after R2 resection was 25 months, significantly shorter than after R0 or R1 resection (115 months; *p* = 0.004). Advanced thymic tumors can be resected with an acceptable risk of complications and low mortality. In stage III as well as in stage IV the promising survival rates are dependent on the resection-status.

## 1. Introduction

In early stages, thymic tumors are limited to the thymus or its surrounding tissue and patients have an excellent prognosis after complete surgical resection [1,2]. In contrast, due to the close anatomical proximity to central structures, advanced diseases are challenging in therapy and require a multidisciplinary approach [3,4]. In 50% of cases these tumors have already invaded the mediastinal tissue at initial presentation. In approximately 30% of these cases, for example, the brachiocephalic vein or the superior vena cava are already involved while in 20% of cases the phrenic nerve is affected. Direct spread to the aorta and pulmonary artery is also seen in one in ten patients [5]. A surgical resection of the tumor still represents the basis of therapy [3]. In addition to surgical treatment, radio-/chemotherapy in adjuvant and neoadjuvant settings as well as local procedures such as hyperthermic intrathoracic chemotherapy (HITOC) are applied In the context of multimodal treatment concepts [3,6]. The prognosis of the patients largely depends on the postoperative resection status, tumor stage, and on the WHO-based histological classification [7]. Tumors of UICC (Union internationale contre le cancer) stage III are characterized by invasion of neighboring structures such as lung, major vessels, phrenic, or vagus nerve [1]. It is further divided into stage IIIa—and thus (still) resectable—tumors and non-resectable stage IIIb tumors [8]. Accordingly, at this stage the mediastinal tumor is resected and relevant structures replaced if necessary [3]. Stage IVa thymic tumors are essentially defined by a pleural dissemination, which has the most relevant effect on therapy [9]. In these patients a pleurectomy/decoration (P/D) of the lung should be performed in addition to the mediastinal tumor resection (unless patients present with an isolated pleural recurrence) which sometimes requires a two-step surgery [3,6]. Positive lymph nodes detected in the histological investigation indicate a stage IVa (N1: anterior region) or IVb (N2: deep region) disease, although their prognostic significance remains still under discussion [1,10,11,12].

The morbidity of these patients is known to be high and due to the low incidence of this rare tumor entity there are limited clinical data available. Surgical treatment of these rare and challenging tumor manifestations is performed in only a few specialized centers worldwide that can fulfill the clinical and technical requirements for such interventions. Therefore, it seems all the more important to share and report clinical experiences and the results of these treatments, which are often made on a case-by-case basis [13,14]. The aim of this study was to compare stage III and IV thymic tumors in terms of performed surgery, completeness of resection, postoperative morbidity, and survival and should therefore provide an overview of the state-of-the-art multimodal treatment of this rare tumor entity.

## 2. Materials and Methods

### 2.1. Study Design

This retrospective, single-center study included all patients who received radical surgical resection of an advanced thymic tumor according to UICC stages III and IV from March 2005 to October 2017 in the thoracic surgery center, Regensburg (Department of Thoracic Surgery, University Medical Centre Regensburg and Department of Thoracic Surgery, Hospital Barmherzige Brueder Regensburg). Approval by our institutional ethics committee was given before retrospective data collection (21-2258-104). Informed patient consent was waived because of the retrospective study design. All patients were recruited in an interdisciplinary tumor board for thoracic oncology assessment. Perioperative data and operative reports were collected from the institutional database and medical records. Routine preoperative staging included computed tomography (CT) imaging of the chest and in selected patients a cine-MRI (magnetic resonance imaging) in order to assess myocardial infiltration or infiltration of the great vessels of the heart. The clinical stage of tumor expansion was determined according to TNM classification which was first described and evaluated by the International Association for the Study of Lung Cancer (IASLC) and the International Thymic Malignancy Interest Group (ITMIG) and then introduced by the UICC in 2014 [8,11,12]. Therefore, since many patients in this study were treated before the introduction of the TNM classification, they were primarily classified according to the commonly used Masaoka-Koga classification. These patients were retrospectively assigned a tumor stage according to UICC on the basis of the existing imaging, surgery, and histopathological reports. Histologic subtype was classified according to the WHO histological classification system [15]. The Clavien-Dindo classification was used to describe postoperative complications [16].

The primary endpoint of the study was the completeness of resection (resection status) depending on the UICC tumor stage. Secondary endpoints included postoperative morbidity, in-hospital mortality, as well as survival analyses.

### 2.2. Therapeutic Approach

The primary aim was a macroscopic complete tumor resection (R0/R1) in all patients. The therapeutic strategy was essentially dependent on preoperative staging and, in particular, on the T-descriptor indicating the extent of local tumor infiltration. If a stage III tumor presented as primarily resectable, primary surgical resection via median sternotomy was usually performed. This surgical treatment of stage III tumors included a radical resection, including the en-bloc resection of the thymoma, along with the thymic gland and perithymic fatty tissue via sternotomy. When required, the resection was also extended to the mediastinal pleura, pericardium, lung, and great vessels. Resected great vessels (superior vena cava, left brachiocephalic vein) were either replaced by ring-augmented PTFE-prosthesis or closed. To ensure complete resection of the tumor, establishment of cardiopulmonary-bypass support without cardiac arrest was necessary in some cases of resection (conventional extracorporal circulation). For this purpose, the ascending aorta and the femoral vein were cannulated, Heparin (350 IE/kg) was administered, and extracorporeal circulation was performed with mild to moderate hypothermia (34 °C to 28 °C).

Patients in stage IV were usually operated on in two sessions. In these cases, the mediastinal component of the tumor was resected in a first operation in accordance with the above-mentioned surgical procedure for stage III tumors. After adequate time for the patient to recover from the first procedure, the pleural manifestation was resected in a second session. The preferred resection of pleural tumors (stage IV) was P/D or extended P/D (eP/D) with resection of the pericardium and/or diaphragm. An extrapleural pneumonectomy (EPP) was only performed if lung-sparing resection was not feasible due to tumor invasion of the lung parenchyma. Patients with stage IVa tumors received HITOC following surgical cytoreduction, depending on their general condition. The thoracic cavity was therefore perfused with cisplatin in a dosage between 100–175 mg/m^2^ body surface area and from March 2014 also with 65 mg doxorubicin for 60 min at 42 °C. Thoracic lymph nodes (N1 and N2) were removed routinely and, if they were histologically positive, the initial stage III changed to stage IVa or IVb depending on the localization. The same applied to the case that fractions of tumors in the lung parenchyma were considered discontinuous and thus metastatic in the pathology report. If radical resection was considered infeasible, margins of the residual tumor areas were clipped in order to facilitate postoperative radiation therapy. In general, patients with pre-existing myasthenia were cared for in an intensive care or intermediate care unit during the first 24 h and preoperative medication for myasthenia were restored orally to patients as early as possible postoperatively. Preoperative cortisone therapy was continued perioperatively with intravenous administration of hydrocortisone.

Multimodality therapy (surgery, chemotherapy, and/or radiotherapy) was administered in patients with advanced stages depending on the exact tumor stage, resection status, and histology. If the tumor did not appear resectable at initial diagnosis, neoadjuvant chemotherapy was administered according to the PAC regime (cisplatin, doxorubicin, cyclophosphamide) or octreotide/prednisolone (patients with octreotide scan-positive staging) in order to improve surgical resectability (partial remission). After re-staging via CT of the chest, surgery was normally scheduled in a period of 3–6 weeks depending on the patient’s general condition and operability. Some patients were presented to us for surgery by external hospitals, so that there could also be delays in the planning of surgery. Adjuvant treatment was administered when appropriate. Reasons for not performing adjuvant therapy were significant comorbidity or patient refusal.

### 2.3. Statistical Analysis

Data collection and statistical analyses were performed using IBM SPSS Statistics, Version 26 (IBM Corporation, Armonk, NY, USA). Categorical data were summarized as absolute numbers and percentages and were compared between groups using the chi-squared test of independence or Fisher’s exact test. Interval scaled data were presented as mean ± SD or as median (IQR) and were compared using Student’s *t*-test or Mann-Whitney U-test depending on the underlying distribution of the variable. The distribution was analyzed using Shapiro-Wilk test. The Kaplan-Meier method was used for the analysis of overall survival (OS) and recurrence/progression-free survival RFS/PFS. Overall survival was calculated from the date of surgery to the time of follow-up (March 2020) or until death of the patient. RFS/PFS was defined as the interval between treatment and the occurrence of a recurrence/progression, death, or last follow-up. The log-rank test was used to examine the influence of the Masaoka-Koga stage and postoperative resection status on survival. A *p*-value < 0.05 was considered statistically significant for all analyses.

## 3. Results

### 3.1. Demographic Data

A total of 73 patients received surgical treatment (Table 1). The mean age of the study population was 53.9 ± 12.6 years and 64.8% (*n* = 40) of patients were male. Nearly half of patients (42.5%; *n* = 31) suffered from myasthenia gravis. With 64.6% (*n* = 47) of patients, the majority of the study population had a primary manifestation of the disease. Of the patients with mediastinal recurrence, 75% (*n* = 6) were in the stage III while 100% (*n* = 18) of those with pleural recurrence were in the group of stage IV. Most frequently diagnosed histological subtypes were WHO B2 with 25 patients (34.2%) and WHO B3 and C with 15 patients (20.5%) each. The proportion of thymic carcinomas in the two groups was balanced (stage III: *n* = 7, 23.3% versus stage IV *n* = 8, 18.6%; *p* = 0.77).

### 3.2. Staging

In the studied period, according to Masaoka-Koga, 74 patients were initially identified in tumor stage III or IV. However, one patient was considered to be classified as stage II according to TNM due to only pericardial involvement. Since, according to this classification, it no longer represented an advanced tumor, the affected patient was excluded from further analyses. A total of 23 patients (31.5%) were assigned to UICC stage IIIa (invasion of the lung, brachiocephalic vein, superior vena cava, chest wall, or phrenic nerve) and seven (9.6%) patients to stage IIIb (infiltration of the aorta, intrapericardial pulmonary artery, myocardium, trachea, or esophagus). Stage IVa is defined by the involvement of anterior perithymic nodes or separate pleural or pericardial nodules and was diagnosed in 35 (47.9%) patients. Stage IVb with involvement of deep intrathoracic or cervical nodes or pulmonary intraparenchymal nodules was observed in eight (11%) patients [8]. The group of patients in stage IVb consisted of three patients in whom tumor in the lung tissue was considered a metastasis (discontinuous growth) in the pathology report, and in five patients tumor was found in deep lymph nodes.

### 3.3. Treatment Data

An induction chemotherapy was performed in 50.7% (*n* = 37) of patients and 6.8% (*n* = 5) had an octreotide therapy preoperative. Of these patients with neoadjuvant therapy, two (4.8%) patients showed progression, 15 (35.7%) patients showed partial remission, and 25 (59.5%) patients had stable disease. Nearly all patients with stage III were operated in one session (96.7%; *n* = 29). The treatment of pleural involvement required a P/D (*n* = 16; 37.2%), eP/D (*n* = 11; 25.6%), and EPP (*n* = 6; 14%). Patients who had not undergone resection of the pleura were either in tumor stage IVb or a planned second operation was not performed (in one case, for example, due to death of the patient). In addition, 25 (58.1%) patients in stage IV received HITOC with cisplatin (*n* = 14; 56%) or cisplatin and doxorubicin (*n* = 11; 44%). Overall, mediastinal pleura was resected in 46 (65.8%), pericardium in 41 (56.2%), and diaphragm in 18 (24.7%) patients. Atypical lung resections were necessary in 33 (45.2%) and anatomic lung resections (segmentectomy, lobectomy) in nine (12.3%) patients.

### 3.4. Postoperative Data

Considering stage III patients only, R0-resection was achieved in 16 (53.3%) patients (Table 2). The proportion of R0-resections was higher in patients with T3 tumor (according UICC) with 60.9% (*n* = 14) than in T4 tumors with 20% R0-resections (*n* = 1; *p* = 0.08). In patients with performed surgical cytoreduction (stage IV), the comparison of R0/R1-resection to R2-resection seems to be especially useful. Rates of macroscopically incompletely (R2-) resected tumors of 23.3% (*n* = 10) were observed.

Most of the postoperative complications required drug therapy or interventions under local anesthesia only (Clavien-Dindo grade 1–3A: *n* = 26, 35.6%). Severe complications (Clavien-Dindo grade 3B-5) occurred in 15 patients (20.5%) (Table 2). A surgical revision was necessary in 13 (17.8%) patients. In stage III there was one case of postoperative bleeding and one case of chylothorax. The remaining revisions were necessary in stage IV patients and included four cases of hematothorax, two cases of pleural empyema, two cases of prolonged parenchymal fistula, and a chylothorax, thrombosis of a prosthesis, and rupture of a diaphragmatic suture. The diagnosis of myasthenia gravis had no statistical influence in terms of the occurrence of postoperative complications (*p* = 0.88). Neither the administration of neoadjuvant octreotide (*p* = 0.38) nor chemotherapy (*p* = 0.48) had a statistically significant impact on the incidence of postoperative complications. Adjuvant chemotherapy was performed in 26 (35.6%) of patients and adjuvant radiotherapy in 24 (32.9%) patients. The hospital mortality was 2.7% (stage III: *n* = 1; stage IVa: *n* = 1) and this rate also represented the 30-day mortality.

### 3.5. Survival Analysis

**Figure 1:**
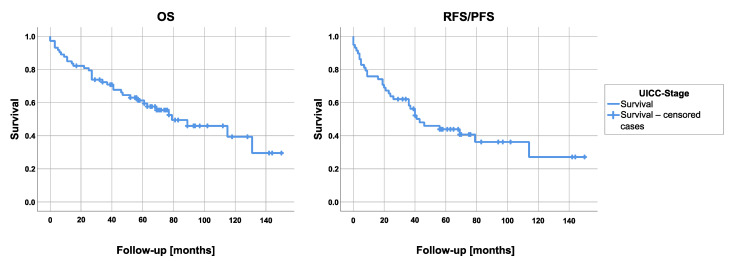
Overall survival (OS) and recurrence/progression-free survival (RFS/PFS).

**Figure 2:**
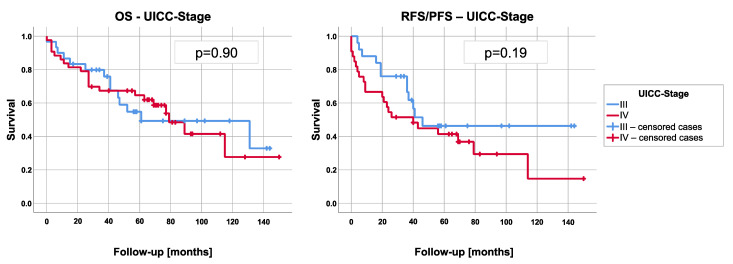
Overall survival (OS) and recurrence/progression-free survival (RFS/PFS) depending on UICC classification.

Data on 73 patients were available for the evaluation of the OS and RFS/PFS. Considering R0 and R1 resected tumors only (*n* = 58), 29 patients (50%) experienced a recurrence or progression of their disease during follow-up (mediastinal *n* = 8, pleural *n* = 12, distant recurrence *n* = 9). The median RFS/PFS was 43 months (95% confidence interval: 22.6–63.5) (Figure 1). With median RFS/PFS of seven (95% confidence interval: 0–33.8) and 20 (95% confidence interval: 0–34.6) months, patients with stage IIIb or IVb thymoma showed a considerably worse RFS/PFS than patients with stage IIIa (median not reached, mean survival 89.7 ± 13.4 months) or IVa (43 months; 95% confidence interval: 0–88.8) tumor (*p* = 0.16). The comparison of stage III versus stage IV results in a median survival of 46 months (95% CI not reached) for stage III patients and 40 months for stage IV patients (95% CI 13.1–66.9), (*p* = 0.19). The corresponding Kaplan-Meier curve is demonstrated in Figure 2. Median OS of all patients was 79 months (95% confidence interval: 40.6–117.4) (Figure 1). The 3- and 5-year survival rates of all patients (stage III and IV) were 72.4% and 61.3%, respectively. Preoperatively diagnosed myasthenia gravis did not affect the patients’ OS (*p* = 0.14). The median OS depending on the UICC classification was 131 (95% confidence interval: 7.4–254.6), 25 (95% confidence interval: 4.5–45.5), 89 (95% confidence interval: 55.4–122.6), and 69 (95% confidence interval: 43.6–94.4) months for stages IIIa, IIIb, IVa, and IVb. Comparing these stages, a significant difference in survival was determined using the log-rank test (*p* = 0.019). In contrast, with a *p*-value of 0.90, the comparison of group III versus IV showed no significant difference in survival (stage III: 61 months (95% CI 0–125.9) versus stage IV: 79 months (95% CI 57.9–100.1) (Figure 2).

**Figure 3:**
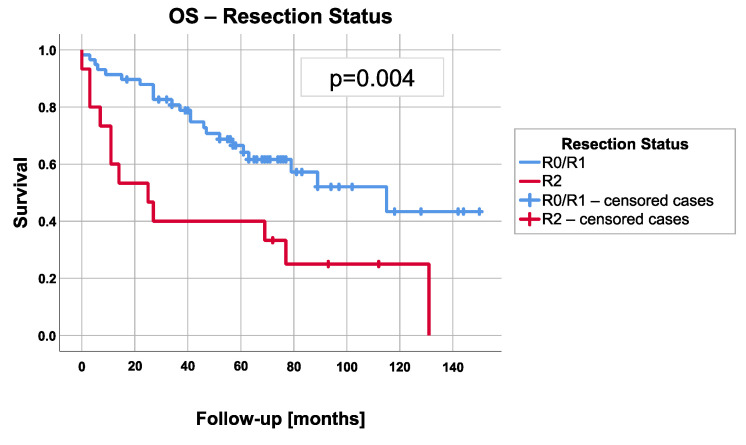
Overall survival (OS) depending on postoperative resection status.

Median OS after R2 resection was 25 months (95% confidence interval 4.8–45.2) and therefore significantly shorter than after R0 or R1 resection with 115 months (95% confidence interval: 60.8–169.2; *p* = 0.004; Figure 3). Overall survival of patients with thymic carcinoma was significantly shorter with a median of 47 months (95% confidence interval 13.3–80.7) compared to 89 months (95% confidence interval 48.6–129.4) in patients with thymoma (*p* = 0.050).

## 4. Discussion

Due to their slow growth and low tendency to metastasize, the prognosis of thymic tumors is generally good—especially when detected in early stages I and II [8]. However, 25% of the patients already present with stage III and 11–12% with stage IV tumors when first diagnosed [17]. The locoregional expansion of the tumor therefore decisively affects therapy and prognosis of the patient [8]. These tumors require a multimodal approach consisting of surgery, radiotherapy and/or chemotherapy [4,18]. Despite the lack of prospective studies, surgical resection still represents the gold standard in the treatment of advanced thymic tumors [19].

Overall, postoperative complications were mostly mild and could be managed conservatively (63.4%). Additionally, the high rate of surgical revisions (17.8%) indicates a high risk of these interventions. There are little data available that specifically describe postoperative morbidity in advanced thymic tumors. Cardillo et al. found a 14.8% rate of major complications in patients with advanced stages of the disease while Yamada et al. reported a 24% rate of postoperative complications in their study population of stage III tumor patients only [20,21]. In their study with patients of all Masaoka-Koga tumor stages, Regnard et al. showed postoperative complication rates of only 8.5%, highlighting how susceptible advanced tumor patients are to severe complications after surgery [7]. We believe that, for this reason, patients in tumor stages III–IV should be treated in experienced centers with an appropriate number of procedures.

The postoperative resection status is known as the most important prognostic factor regarding survival [7,22,23]. The rate of R0 resection was 53% in our stage III patients. Thus, our results are similar to those in the literature, where rates of postoperative R0-status range from 50–80% [24,25,26]. Compared to the Masaoka-Koga classification, the UICC-/TNM classification defines even more specifically the borderline between primary resectable tumors or the indication for previous induction therapy with stages IIIA and IIIB (*p* = 0.08) [8]. In tumor stage IVa, a classification as R0 does not appear to be appropriate, since it is known well that residual tumor cells remain in the thoracic cavity after surgery due the disseminated growth pattern. It often is technically only possible to achieve macroscopic tumor removal; secondly, it is difficult for the pathologist to classify the histological material as R0. However, a macroscopic complete (R0/R1) resection could be achieved in most cases (80%) of our study. In analogy to the surgical therapy of malignant pleural mesothelioma, these operations (P/D, eP/D, EPP) are also referred to as surgical cytoreduction [27]. The benefit of this approach for thymic tumors is the subject of controversial discussions. While some investigations consider the advantage of aggressive tumor debulking to be merely a reduction of the field to be irradiated afterwards, other, mostly retrospective, studies suggested a survival advantage by this approach [28,29,30]. For mediastinal tumors, we regularly performed intraoperative frozen section examinations when resection margins were unclear. In some cases, however, we encountered inevitable R1/R2-situations with no further options for resection, so that markings were made to facilitate postoperative radiotherapy. In our study, for example, the patients in stage III showed very good survival prognoses despite only about 50% R0-resections, thus seeming to have possibly benefited from tumor debulking.

The survival of patients with advanced thymic tumors depends on numerous factors. In our cohort, 87.6% of the patients had a tumor histology of WHO B2 or worse, which is often the case in the advanced stages examined here [31]. It is well known that this is also of prognostic relevance since thymic carcinomas (WHO C) demonstrate a substantially more aggressive growth and hence the survival of patients suffering from this tumor entity is limited [31,32]. As expected, overall survival of patients with thymic carcinoma was also significantly worse than those with thymoma in our study (47 months versus 89 months, *p* = 0.050). However, the proportion of thymic carcinomas in the examined stage III and stage IV study groups was balanced around 20% each.

This study is focused on the impact of the tumor stage and the postoperative resection status on the further course of disease. First of all, it has to be stated that the median OS of 79 months of patients in advanced stages III and IV clearly differs from the excellent survival rates of lower tumor stages reported in the previous literature. For example, Regnard et al. demonstrated 15-year survival rates of 78% and 73% for stage I and II thymic tumor patients. Our corresponding 5-year survival rate of 61.3% can surely indicate the difference. Another work with advanced tumors was published by Cardillo et al., who reported an overall 10-year survival rate of 50.6%. However, they had no patients with the highest tumor stage IVb in their cohort [20]. With regard to tumor stages, however, we could not prove a statistically significant difference in RFS/PFS (*p* = 0.19) or OS (*p* = 0.90). Accordingly, higher tumor stage is not necessarily associated with worse survival in our study, as for example pleural involvement (stage IVa, Median OS 89 months) apparently can be survived longer than an involvement of essential mediastinal structures (stage IIIb, Median OS 25 months). Only the postoperative resection status proved to be statistically significant prognostic factor regarding the OS (*p* = 0.005).

For elimination of the mentioned residual tumor cells in patients with pleural involvement, additional procedures such as HITOC can be applied. The ESMO Clinical Practice Guidelines for diagnosis, treatment, and follow-up of thymic epithelial tumors states that hyperthermic intrapleural chemotherapy may be discussed in case of stage IVa tumors. However, only retrospective cohort studies are available for this procedure (level of recommendation: C) [6]. With 71.4%, most of our stage IVa patients were treated with this additional procedure. Another study of our working group analyzed stage IVa thymoma patients with subsequent HITOC and showed an overall 5-year survival rate of 80.1% [32]. The comparison with the 5-year survival rate of 61.8% determined in this study indicates that patients in this stage may show a better survival under adequate therapy than those in lower tumor stages. In our opinion, it also shows that HITOC is an important component in multimodal therapy in patients with pleural involvement that can improve survival considerably and which has to be analyzed in future studies.

Limitations of this study are its retrospective nature and the non-randomized, single-center design. In addition, our cohort was acquired over a period of more than ten years, so that treatment modalities were evolving during this time and are therefore not uniform. Meanwhile, the UICC-stages increasingly replaced the Masaoka-Koga classification. The original histopathological reports mostly used the Masaoka-Koga classification. We used the UICC-stages for this retrospective analysis, which were defined according to imaging, surgery, and pathology reports. In addition, it should be mentioned that the groups of tumor stages IIIb and IVb were especially small, with seven and eight patients, respectively, so that further adjustment of the analyses regarding possible cofactors was statistically not feasible.

## 5. Conclusions

Advanced thymic tumors can be resected with an acceptable risk of major complications and low mortality. Significant differences of the tumor stages regarding recurrence/progression-free or overall survival were not found. In stage III as well as in stage IV the promising survival rates are mainly dependent on the resection status.

## Figures and Tables

**Table 1:** Patients’ characteristics.

Title	Study Population (*n* = 73; 100%)
Mean Age ± SD [years]	53.9 ± 12.6
Male Gender; *n* (%)	40 (54.8)
Tumor manifestation; *n* (%)	
Initial manifestation	47 (64.4)
Mediastinal recurrence	8 (11)
Pleural recurrence	18 (24.7)
Histologic Subtype; *n* (%)	
WHO A	1 (1.4)
WHO AB	2 (2.7)
WHO B1	6 (8.2)
WHO B2	25 (34.2)
WHO B2/B3	9 (12.3)
WHO B3	15 (20.5)
WHO C	15 (20.5)
UICC-Stage; *n* (%)	
IIIa	23 (31.5)
IIIb	7 (9.6)
IVa	35 (47.9)
IVb	8 (11)

SD = standard deviation, UICC = Union internationale contre le cancer, WHO = World Health Organization.

**Table 2:** Postoperative data.

Title	All Patients (*n* = 73; 100%)	UICC Stage III (*n* = 30; 100%)	UICC Stage IV (*n* = 43; 100%)	*p*-Value
Resection status; *n* (%)				
– R0	27 (37)	16 (53.3)	11 (25.5)	0.052
– R1	31 (42.5)	9 (30)	22 (51.2)	
– R2	15 (20.5)	5 (16.7)	10 (23.3)	
Postoperative complications; *n* (%)	41 (56.2)	13 (43.3)	28 (65.1)	
– Grade 1	7 (9.6)	1 (3.3)	6 (14)	0.15
– Grade 2	14 (19.2)	7 (23.3)	7 (16.3)	
– Grade 3A	5 (6.8)	2 (6.6)	3 (7)	
– Grade 3B	13 (17.8)	2 (6.6)	11 (25.6)	
– Grade 4A	0 (0)	0 (0)	0 (0)	
– Grade 4B	0 (0)	0 (0)	0 (0)	
– Grade 5	2 (2.7)	1 (3.3)	1 (2.3)	
Surgical revision; *n* (%)	13 (17.8)	2 (6.6)	11 (25.6)	0.038
Adjuvant therapy; *n* (%)				
– Chemotherapy	26 (35.6)	7 (23.3)	19 (44.2)	0.10
– Radiotherapy	24 (32.9)	14 (46.7)	10 (23.3)	0.020
In-hospital mortality; *n* (%)	2 (2.7)	1 (3.3)	1 (2.3)	0.80

## Data Availability

The data presented in this study are available on request from the corresponding author.

## References

[1] Koga K., Matsuno Y., Noguchi M., Mukai K., Asamura H., Goya T., Shimosato Y. (1994). A review of 79 thymomas: Modification of staging system and reappraisal of conventional division into invasive and non-invasive thymoma. Pathol. Int..

[2] Safieddine N., Liu G., Cuningham K., Ming T., Hwang D., Brade A., Bezjak A., Fischer S., Xu W., Azad S. (2014). Prognostic factors for cure, recurrence and long-term survival after surgical resection of thymoma. J. Thorac. Oncol..

[3] Ried M., Guth H., Potzger T., Diez C., Neu R., Schalke B., Hofmann H.S. (2012). Surgical resection of thymoma still represents the first choice of treatment. Thorac. Cardiovasc. Surg..

[4] Venuta F., Anile M., Diso D., Vitolo D., Rendina E.A., De Giacomo T., Francioni F., Coloni G.F. (2010). Thymoma and thymic carcinoma. Eur. J. Cardiothorac. Surg..

[5] Detterbeck F.C., Zeeshan A. (2013). Thymoma: Current diagnosis and treatment. Chin. Med. J..

[6] Girard N., Ruffini E., Marx A., Faivre-Finn C., Peters S., Committee E.G. (2015). Thymic epithelial tumours: ESMO Clinical Practice Guidelines for diagnosis, treatment and follow-up. Ann. Oncol..

[7] Regnard J.F., Magdeleinat P., Dromer C., Dulmet E., de Montpreville V., Levi J.F., Levasseur P. (1996). Prognostic factors and long-term results after thymoma resection: A series of 307 patients. J. Thorac. Cardiovasc. Surg..

[8] Detterbeck F.C., Stratton K., Giroux D., Asamura H., Crowley J., Falkson C., Filosso P.L., Frazier A.A., Giaccone G., Huang J. (2014). The IASLC/ITMIG Thymic Epithelial Tumors Staging Project: Proposal for an evidence-based stage classification system for the forthcoming (8th) edition of the TNM classification of malignant tumors. J. Thorac. Oncol..

[9] Ried M., Eicher M.M., Neu R., Sziklavari Z., Hofmann H.S. (2017). Evaluation of the new TNM-staging system for thymic malignancies: Impact on indication and survival. World J. Surg. Oncol..

[10] Viti A., Bertolaccini L., Terzi A. (2014). What is the role of lymph nodal metastases and lymphadenectomy in the surgical treatment and prognosis of thymic carcinomas and carcinoids?. Interact. Cardiovasc. Thorac. Surg..

[11] Bhora F.Y., Chen D.J., Detterbeck F.C., Asamura H., Falkson C., Filosso P.L., Giaccone G., Huang J., Kim J., Kondo K. (2014). The ITMIG/IASLC Thymic Epithelial Tumors Staging Project: A Proposed Lymph Node Map for Thymic Epithelial Tumors in the Forthcoming 8th Edition of the TNM Classification of Malignant Tumors. J. Thorac. Oncol..

[12] Kondo K., Van Schil P., Detterbeck F.C., Okumura M., Stratton K., Giroux D., Asamura H., Crowley J., Falkson C., Filosso P.L. (2014). The IASLC/ITMIG Thymic Epithelial Tumors Staging Project: Proposals for the N and M components for the forthcoming (8th) edition of the TNM classification of malignant tumors. J. Thorac. Oncol..

[13] Gadalla S.M., Rajan A., Pfeiffer R., Kristinsson S.Y., Bjorkholm M., Landgren O., Giaccone G. (2011). A population-based assessment of mortality and morbidity patterns among patients with thymoma. Int. J. Cancer.

[14] Ried M., Neu R., Schalke B., von Susskind-Schwendi M., Sziklavari Z., Hofmann H.S. (2015). Radical surgical resection of advanced thymoma and thymic carcinoma infiltrating the heart or great vessels with cardiopulmonary bypass support. J. Cardiothorac. Surg..

[15] Travis W.D., Brambilla E., Burke A.P., Marx A., Nicholson A.G. (2015). WHO Classification of Tumours of the Lung, Pleura, Thymus and Heart.

[16] Dindo D., Demartines N., Clavien P.A. (2004). Classification of surgical complications: A new proposal with evaluation in a cohort of 6336 patients and results of a survey. Ann. Surg..

[17] Detterbeck F.C., Parsons A.M. (2011). Management of stage I and II thymoma. Thorac. Surg. Clin..

[18] Aprile V., Bacchin D., Korasidis S., Nesti A., Marrama E., Ricciardi R., Petrini I., Ambrogi M.C., Paladini P., Lucchi M. (2020). Surgical treatment of pleural recurrence of thymoma: Is hyperthermic intrathoracic chemotherapy worthwhile?. Interact. Cardiovasc. Thorac. Surg..

[19] Davenport E., Malthaner R.A. (2008). The role of surgery in the management of thymoma: A systematic review. Ann. Thorac. Surg..

[20] Cardillo G., Carleo F., Giunti R., Lopergolo M.G., Salvadori L., De Massimi A.R., Petrella L., Martelli M. (2010). Predictors of survival in patients with locally advanced thymoma and thymic carcinoma (Masaoka stages III and IVa). Eur. J. Cardiothorac. Surg..

[21] Yamada Y., Yoshino I., Nakajima J., Miyoshi S., Ohnuki T., Suzuki M., Nagayasu T., Iwasaki A., Okumura M., Japanese Association for Research of the Thymus (2015). Surgical Outcomes of Patients with Stage III Thymoma in the Japanese Nationwide Database. Ann. Thorac. Surg..

[22] Ahmad U., Yao X., Detterbeck F., Huang J., Antonicelli A., Filosso P.L., Ruffini E., Travis W., Jones D.R., Zhan Y. (2015). Thymic carcinoma outcomes and prognosis: Results of an international analysis. J. Thorac. Cardiovasc. Surg..

[23] Scorsetti M., Leo F., Trama A., D’Angelillo R., Serpico D., Macerelli M., Zucali P., Gatta G., Garassino M.C. (2016). Thymoma and thymic carcinomas. Crit. Rev. Oncol. Hematol..

[24] Detterbeck F.C., Parsons A.M. (2004). Thymic tumors. Ann. Thorac. Surg..

[25] Wright C.D. (2010). Extended resections for thymic malignancies. J. Thorac. Oncol..

[26] Spaggiari L., Leo F., Veronesi G., Solli P., Galetta D., Tatani B., Petrella F., Radice D. (2007). Superior vena cava resection for lung and mediastinal malignancies: A single-center experience with 70 cases. Ann. Thorac. Surg..

[27] Ruffini E., Filosso P.L., Guerrera F., Lausi P., Lyberis P., Oliaro A. (2018). Optimal surgical approach to thymic malignancies: New trends challenging old dogmas. Lung Cancer.

[28] Curran W.J., Kornstein M.J., Brooks J.J., Turrisi A.T. (1988). Invasive thymoma: The role of mediastinal irradiation following complete or incomplete surgical resection. J. Clin. Oncol..

[29] Lin C.S., Kuo K.T., Hsu W.H., Huang B.S., Wu Y.C., Hsu H.S., Huang M.H., Wang L.S. (2004). Managements of locally advanced unresectable thymic epithelial tumors. J. Chin. Med. Assoc..

[30] Liu H.C., Chen Y.J., Tzen C.Y., Huang C.J., Chang C.C., Huang W.C. (2006). Debulking surgery for advanced thymoma. Eur. J. Surg. Oncol..

[31] Detterbeck F., Youssef S., Ruffini E., Okumura M. (2011). A review of prognostic factors in thymic malignancies. J. Thorac. Oncol..

[32] Markowiak T., Neu R., Ansari M.K.A., Grosser C., Klinkhammer-Schalke M., Hofmann H.S., Ried M. (2019). Surgical Cytoreduction and HITOC for Thymic Malignancies with Pleural Dissemination. Thorac. Cardiovasc. Surg..

